# Pyruvate Might Bridge Gut Microbiota and Muscle Health in Aging Mice After Chronic High Dose of Leucine Supplementation

**DOI:** 10.3389/fmed.2021.755803

**Published:** 2021-11-22

**Authors:** Yuxiao Liao, Dan Li, Xiaolei Zhou, Zhao Peng, Zitong Meng, Rui Liu, Wei Yang

**Affiliations:** ^1^Department of Nutrition and Food Hygiene, Hubei Key Laboratory of Food Nutrition and Safety, Tongji Medical College, Huazhong University of Science and Technology, Wuhan, China; ^2^Department of Nutrition and Food Hygiene and MOE Key Lab of Environment and Health, School of Public Health, Tongji Medical College, Huazhong University of Science and Technology, Wuhan, China; ^3^Department of Preventive Medicine, School of Medicine, Jianghan University, Wuhan, China

**Keywords:** leucine, muscle health, gut microbiota, aging mice, pyruvate

## Abstract

**Background:** The previous studies demonstrated that there might be complex and close relationships among leucine supplementation, gut microbiota, and muscle health, which still needs further investigation.

**Aims:** This study aimed to explore the associations of gut microbiota with muscle health after leucine intake.

**Methods:** In this study, 19-month-old male C57BL/6j mice (*n* = 12/group) were supplemented with ultrapure water, low dose of leucine (500 mg/kg·d), and high dose of leucine (1,250 mg/kg·d) for 12 weeks by oral gavage. The mice fecal samples in each group before and after supplementation were collected for baseline and endpoint gut microbiota analysis by using 16S rDNA amplicon sequencing. Meanwhile, ultrasound measurement, H&E staining, myofiber cross-sectional area (CSA) measurement, and western blotting were performed in the quadriceps subsequently. The pyruvate levels were detected in feces.

**Results:** Improvement in muscle of histology and ultrasonography were observed after both low and high dose of leucine supplementation. High dose of leucine supplementation could promote skeletal muscle health in aging mice *via* regulating AMPKα/SIRT1/PGC-1α. The richness and diversities of microbiota as well as enriched metabolic pathways were altered after leucine supplementation. *Firmicutes*-*Bacteroidetes* ratio was significantly decreased in high-leucine group. Moreover, pyruvate fermentation to propanoate I were negatively associated with differential species and the pyruvate levels were significantly increased in feces after high dose of leucine supplementation.

**Conclusions:** Chronic high dose of leucine supplementation changed gut microbiota composition and increased pyruvate levels in the feces, which possibly provides a novel direction for promoting muscle health in aging mice.

## Introduction

Muscle aging is characterized by decline in muscle mass and function as well as increase in fat mass with growing age, consequently leading to age-related sarcopenia, if without any preventive timely treatment (1, 2). Astonishingly, muscle mass and function begins to decline approximately at the age of 30 years, and rates of decline gradually accelerate as age increases (3), which would be aggravated by increased fat mass in muscle. The risks of falls, frailty, loss of ability to perform basic activities, type-II diabetes and cognitive performance decline were increased in elderly people with muscle aging, which all impaired the quality of life and caused premature mortality (4, 5). Moreover, a research based on the National Health and Nutrition Examination Survey (NHANES) demonstrated that muscle mass is a significant predictor of longevity for all-cause mortality in population of men and women aged over 55 and 65, respectively (6). Hence, it is urgent to explore an effective way to promote muscle health in the aging people.

Nutritional supplementation, such as protein and branched chain amino acids (BCAAs), has provided an easy and efficient way to promote muscle health for years. Leucine, an essential BCAA, was widely demonstrated to advance muscle health in the model of old animal and humans (7). Both our published reviews and other studies revealed that leucine intake could promote muscle protein synthesis and improve myofiber regeneration by mammalian target of rapamycin (mTOR) signaling pathway in the elderly (2, 8–10). Notably, leucine was reported to modulate energy homeostasis by activating energy sensor network, AMP-activated protein kinase (AMPK)/silent information regulator 1 (SIRT1)/peroxisome proliferator-activated receptor γ co-activator 1α (PGC-1α) (11). However, whether long-term leucine intake could impact on muscle health in old mice *via* regulating AMPKα/SIRT1/PGC-1α remains largely uncertain.

In recent years, a growing focus on the impact of BCAAs, such as leucine, on the composition and function of gut microbiota, consequently making difference on health. BCAAs balance markedly improved *Gammaproteobacteria, Lactobacillales*, and *Aeromonadales* proliferation in piglets, which might mediate growth promotion and amino acid metabolism (12). Leucine concentration in serum was reported to correlate positively with the abundance of *Bacteroides* in postmenopausal women (13). Leucine intake enhanced intestinal health through regulation of mTOR pathway and promoting SIgA secretion in 6-week-old mice intestine, which might be linked with markedly and shifted *Firmicutes*-*Bacteroidetes* ratio (14). Another study demonstrated that leucine supplementation decreased body fat weight in pigs, associated with higher *Actinobacteria* and increased colonic butyrate and propionate concentrations (15). Moreover, Zhang *et al*. summarized that leucine may regulate lipid metabolism by modulating gut microbiota and short-chain fatty acids (11). Thus, given that leucine intake had well-known impact on muscle health, investigation about the effects of leucine on gut microbiota in an aging animal model is essential.

Gut microbiota would change along with aging, which were associated with the physiological decline of musculoskeletal function of the host (16). A report revealed that aging skewed the composition of the gut microbiome particularly by altering the *Sutterella* to *Barneseilla* ratio and altered the metabolic potential of intestinal bacteria by comparing the gut microbiota of aged sarcopenic rats (8, 18, and 24 months) (17). Moreover, the microbiota may underlie the sarcopenic phenotype of the aged rats *via* vitamin synthesis, altered lipid metabolism, and regulation of growth and immune-related factors, as a previous study also suggested the same (17). Roger *et al*. transplanted microbiota of high-physical-functioning older adults (70–85 years old) to germ-free mice and demonstrated that gut microbiota may play a role in maintenance of muscle strength (18). However, whether gut microbiota would participate in the effects of leucine on muscle health in aging mice is still unknown.

As mentioned above, there are complex and close relationships among leucine intake, gut microbiota, and muscle health in an aging animal model. Hence, in this exploratory study, 19-month-old mice were supplemented by low and high dose of leucine (500 and 1250 mg/kg·d) for 12 weeks to explore the chronic effects of leucine on muscle health. Simultaneously, the mice feces were collected before and after leucine supplementation for microbiota analysis, for the sake of studying the possible association of gut microbiota with muscle health after leucine intake in aging mice.

## Materials and Methods

### Animals

Nineteen-month-old male C57BL/6j mice were purchased from Vital River Laboratory Animal Technology Company (Beijing, China) and housed in specific pathogen-free animal laboratory (one mouse per cage) with controlled temperature (23 ± 2°C), relative humidity (55 ± 5%), 12-h light/dark cycle, and ventilation (air exchange rate of 18 times per hour). The mice were allowed *ad libitum* access to food and water. The study was approved by the Institutional Animal Care and Use Committee of Tongji Medical College, Huazhong University of Science and Technology (IACUC number: S407) in line with the guidelines of National Institute of Health Guide for the Care and Use of Laboratory Animals.

### Experiment Design

It is widely-accepted that 500 mg/kg·d was the upper limit for leucine in both young and old men, while 1,250 mg/kg·d was investigated to be the maximum safe level of intake in young men, since temporary hyperammonemia would appear but no other adverse effects were observed at these two doses (19–21). Quadriceps strength would obviously decrease by 3–5% per year in 60-year-old men (3), whose age was equivalent to mice around 19 months according to The Jackson Laboratory. Additionally, most of the reported leucine intake studies were short term, instead of long term (19). Hence, we applied 500 and 1,250 mg/kg·d as low and high leucine supplementation levels in 19-month-old mice for 12 weeks, to investigate the chronic effects of leucine intake on muscle health in an aging animal model.

A sample size of 36 mice (12 mice/group) were selected by referring to the previous similar papers (22–24) as well as following the 4R principles (25). This study is a regular nutrition intervention experiment on healthy aging mice, which has no specific requirement on animal model. Hence, no specific criteria for including and excluding animals were set in this study. Randomization was carried out as follows. After 1-week adaptive feeding, a total number of 36 mice were weighed and reordered from high to low body weight. Three cages were labeled A, B, and C, respectively. Starting from mouse with the highest body weight, the mice were allocated into each cage successively according to the order of body weight. The first round, three mice were allocated into A, B, and C cage successively. The second round, three mice were allocated into B, C, and A cage successively. The third round, three mice were allocated into C, A, and B cage successively, and so on, until all mice were allocated into the cages (12 mice per cage). Cages A, B, and C were designated as control, low-leucine, and high-leucine group (*n* = 12/group), respectively. Each mouse in each group was raised in one cage alone.

**Figure 1:**
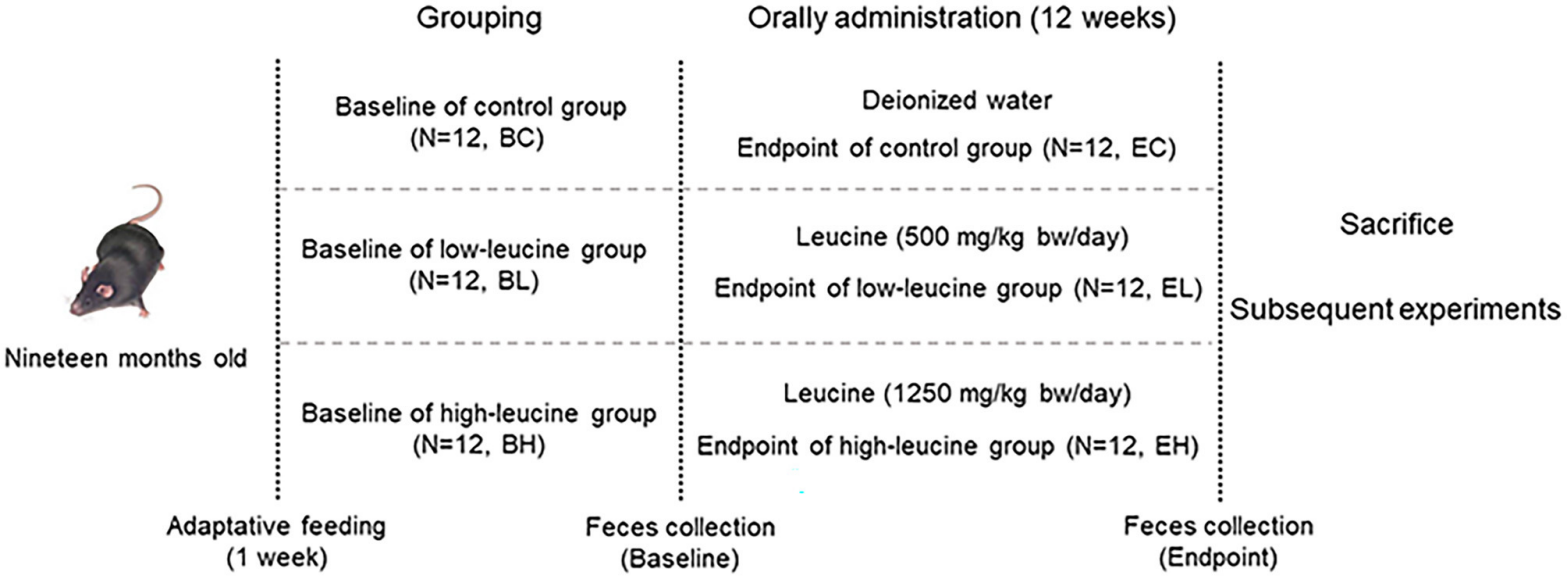
Experiment design scheme.

The mice feces of three groups were collected for the baseline microbiota analysis before gavage (baseline groups: baseline of control (BC) group, baseline of low-leucine (BL) group, and baseline of high-leucine (BH) group). Mice were administered by L-Leucine (powder dissolved in ultrapure water, BioFRoxx, 1215GR500, purity ≥ 99%, Germany) once a day by oral gavage at doses of 500 mg/kg body mass and 1,250 mg/kg body mass in low-leucine and high-leucine groups for 12 weeks, while control group received ultrapure water only. After gavage, the mice feces were also collected in three groups for endpoint microbiota analysis (endpoint groups: endpoint of control (EC) group, endpoint of low-leucine (EL) group, and endpoint of high-leucine (EH) group). All the fecal samples were obtained and immediately stored at −80°C until analysis. Finally, the mice quadriceps were collected and stored at −80°C after the sacrifice of mice (experiment design shown in Figure 1).

### H&E Staining

The quadriceps were fixed in 4% paraformaldehyde for 24 h at least, then, embedded in paraffin blocks, and cross-sectioned at a thickness of 4 μm (Leica, Solms, Germany). The sections were stained with H&E for morphological analysis. Quadriceps microscopy was performed using an Olympus IX-71 microscope (Tokyo, Japan).

### Muscle Fiber Cross-Sectional Area (CSA) Measurements

Six images of different locations in one H&E section were captured at × 200 magnification for muscle fiber CSA calculation. Fifty contiguous myofibers in each image were circled to obtain an average of 300 fibers for each muscle. Image J software (National Institutes of Health, Bethesda, MD, USA) was used to determine the area and number of muscle fibers.

### Ultrasound Measurement in Quadriceps

The ultrasonography was used to evaluate muscle mass by measuring muscle thickness. Muscle thickness was determined using a B-mode ultrasound system (ACUSON S3000; SIEMENS, CA, USA) with a 10.0-MHz transducer (ACUSON 18L6 HD; SIEMENS, CA, USA). Mice were sedated with a subcutaneous injection of 250 mg/kg tribromoethanol. The hair on the right femoral external side was clipped. The transducer with a generous amount of gel was placed perpendicular to the long axis of the femur or muscle. Quadriceps ultrasound was performed after leucine gavage.

### DNA Extraction and 16S rRNA Gene Sequencing

Total microbial DNA was extracted from the baseline and endpoint mice feces in each group (*n* = 9/group) using the QIAamp Fast DNA Stool Mini Kit (Qiagen, Germany), and DNA quality control was preformed using Thermo Nanodrop 2000 fluorometer (Thermo Fisher Scientific, MA, USA) and 1% agarose gel electrophoresis. PCR was performed to produce V3-V4 hypervariable regions of the 16S rRNA gene using the conserved primers 341F (5′-CCTACGGGRSGCAGCAG-3′) and 806R (5′-GGACTACVVGGGTATCTAATC-3′). The PCR fragments were sequenced with Illumina Miseq PE250 by Realbio Technology (Shanghai, China) under standard instruction. The sequencing data analysis was performed following methods in Supplementary Materials.

### Pyruvate Detection

The levels of pyruvate of three groups in the mice feces before leucine gavage, and in the feces and quadriceps after leucine gavage were detected by pyruvate assay kits (E-BC-K130-M, Elabscience, Wuhan, China) respectively, following the instructions of manufacturer. The results of pyruvate levels were presented as μmol/ml in per mg feces.

### Western Blotting

Total protein was extracted from mice quadriceps. After being separated with 10% sodium dodecyl sulfate-PAGE (SDS-PAGE), the proteins were transferred to nitrocellulose membranes. The proteins were probed with primary antibodies against AMPKα (#5831, 1:1000, Cell Signaling Technology, MA, USA), p-AMPKα (thr172) (#2535, 1:1000, Cell Signaling Technology, MA, USA), SIRT1 (#9475, 1:1000, Cell Signaling Technology, MA, USA), PGC-lα (ab54481, 1:1000, Abcam, UK), PPARγ (#A0270,1:1000, ABclonal, Wuhan, China), Foxo3a (#2497, 1:1000, Cell Signaling Technology, MA, USA), MEF2C (#5030, 1:1000, Cell Signaling Technology, MA, USA), FASN (A0461, 1:1000, ABclonal, Wuhan, China), Atrogin-1 (sc-166806-HRP, 1:1000, Santa Cruz, USA), Myod1 (A16218, 1:1000, ABclonal, Wuhan, China), and GAPDH (#5174, 1:10000, Cell Signaling Technology, MA, USA). Secondary HRP-linked antibody (#7076, 1:10000, Cell Signaling Technology, MA, USA) and Lumigen ECL Ultra (Lumigen, MI, USA) detection reagents were used to visualize proteins.

### Statistical Analysis

Data are expressed as the mean ± SEM of at least three independent experiments for each group. One-way ANOVA was used to evaluate the data for comparisons between the two groups and multiple groups, respectively. A statistical analysis was conducted and graphed by GraphPad Prism (Version 8.0, CA, USA). A value of *P* < 0.05 was considered statistically significant.

## Results

### Improvement in Muscle of Histology and Ultrasonography Were Observed After Leucine Supplementation

**Figure 2:**
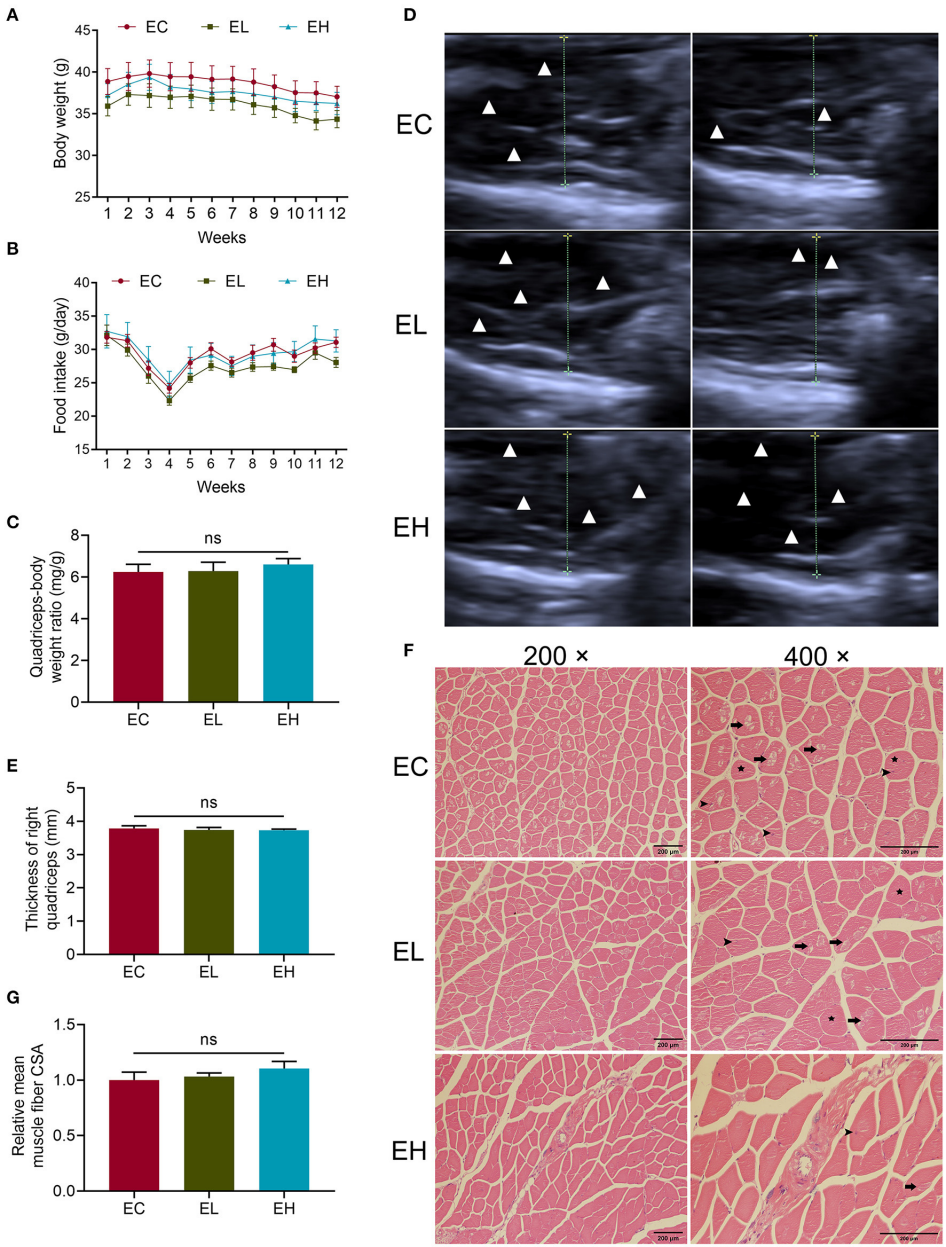
Phenotype changes after leucine supplementation in aging mice. **(A,B)** Body weight and food intake variation throughout the experiment; **(C)** quadriceps-body weight ratio in endpoint groups; **(D,E)** the quadriceps ultrasonographic images as well as muscle thickness of right quadriceps in endpoint groups. Muscle fiber appeared hypoechoic, while perimysium and fascia appeared hyperechoic. White triangles represented hypoechoic areas while the dotted lines showed the transverse diameters of quadriceps; **(F)** H&E staining sections of quadriceps in endpoint groups, scale bar: 200 μm. Long arrows pointed to vacuolization, stars represented rounding and short arrows pointed to nuclei ingression in muscle fibers. **(G)** Relative mean muscle fiber cross-sectional area (CSA) in the endpoint groups. ns, no significance.

Body weight gain was similar in the three groups throughout the experiment period (*P* > 0.05, Figure 2A). Likewise, food intake was analyzed throughout the experiment and did not differ among the three groups (*P* > 0.05, Figure 2B). To evaluate the effects of leucine supplementation for 12 weeks on muscle mass, quadriceps-body weight ratio comparison was made among the three groups. However, there was no significant difference of quadriceps-body weight ratio among the three groups (*P* > 0.05, Figure 2C). Ultrasonography of Quadriceps was also used to characterize muscle thickness in the three groups. Muscle fiber appeared hypoechoic, while perimysium and fascia appeared hyperechoic. As ultrasonic graphs showed, there were obviously more hypoechoic areas as doses of leucine supplementation increased (Figure 2D). Nevertheless, thickness of right quadriceps was also not significantly different among the three groups (*P* > 0.05, Figure 2E). Otherwise, the H&E staining sections displayed there are more evident muscle fibers vacuolization, rounding, nuclei ingression in EC group, indicating leucine could lay promoting effects on the histology of quadriceps tissues in the EL and EH groups (Figure 2F). The mean muscle fiber CSA was measured to evaluate muscle fiber size in the three groups according to the H&E section images, whereas, mean CSA had no significant difference among the three groups (*P* > 0.05, Figure 2G).

### Chronic High Dose of Leucine Supplementation Could Impact on Skeletal Muscle Health *via* Regulating AMPKα/SIRT1/PGC-1α in Aging Mice

**Figure 3:**
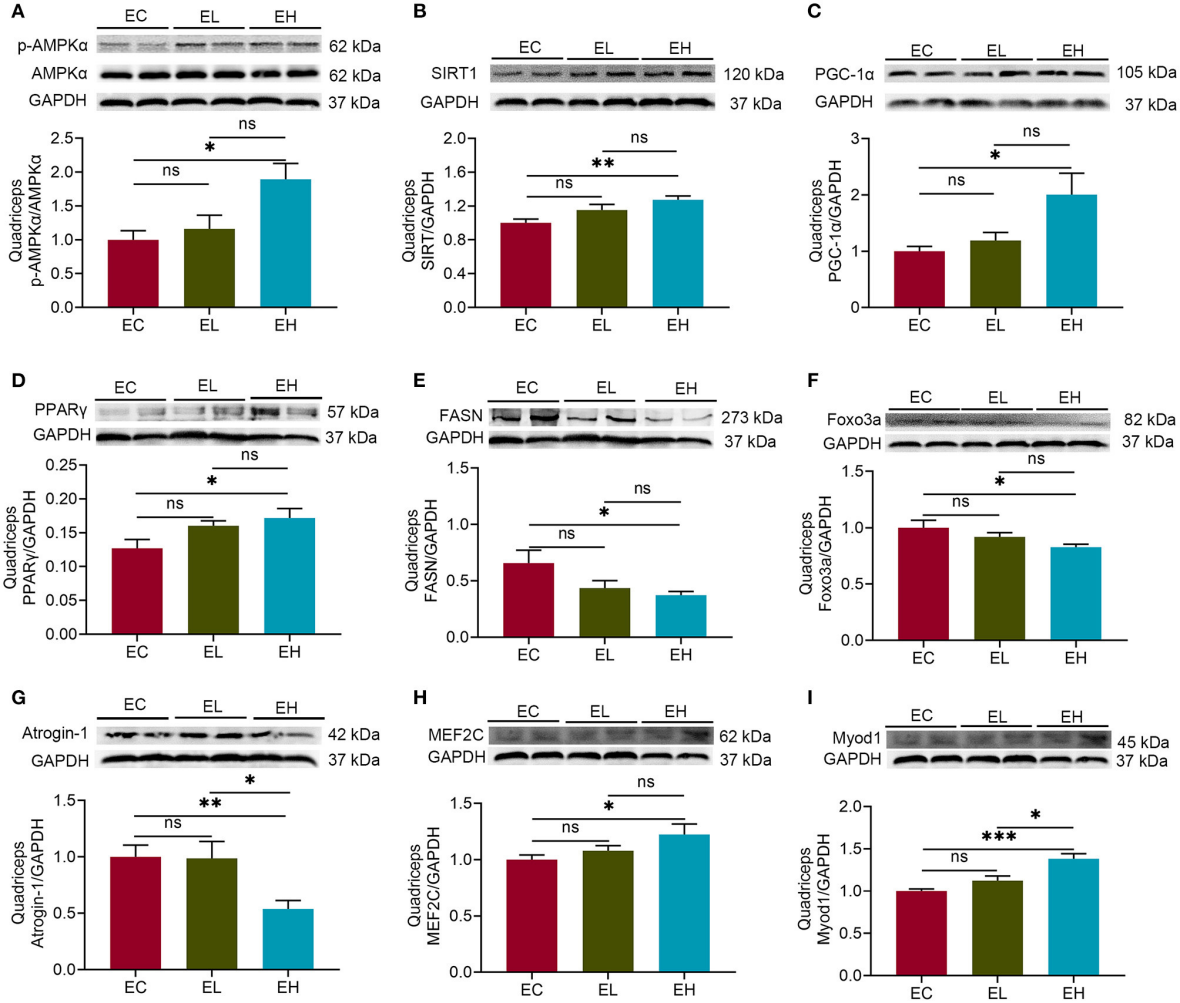
Leucine supplementation could impact on skeletal muscle health *via* regulating AMPKα/SIRT1/PGC-1α in aging mice. **(A)** Protein levels of p-AMPKα (thr172)/AMPKα in the endpoint groups; **(B,C)** the protein levels of SIRT1 and PGC-lα in the endpoint groups; **(D,E)** the protein levels of peroxisome proliferator-activated receptor γ (PPARγ) and fatty acid synthase (FASN) in the endpoint groups; **(F,G)** the protein levels of fork head box protein O 3a (Foxo3a) and Atrogin-1 in the endpoint groups; **(H,I)** the protein levels of myocyte enhancer factor-2C (MEF2C) and myogenic differentiation 1 (MyoD1) in the endpoint groups; ns, no significance; *P < 0.05; **P < 0.01; and ***P < 0.001.

To explore whether leucine supplementation could exert the effects on skeletal muscle in aging mice through AMPK/SIRT1/PGC-1α, protein levels of AMPKα, SIRT1, and PGC-lα in quadriceps were measured by using western-blot. There was significantly higher p-AMPKα (thr172)/AMPKα in EH group, compared with that in EC group (*P* < 0.05, Figure 3A), suggesting that AMPKα pathway was activated by leucine supplementation. The protein levels of both SIRT1 and PGC-lα were significantly increased in EH group compared with that in EC group (*P* < 0.05, Figures 3B,C).

In addition, PGC-1α, acts as a myokine controller, is known to interact with several nuclear transcription factors, such as peroxisome proliferator-activated receptor γ (PPARγ), fork head box protein O 3a (Foxo3a), and myocyte enhancer factor-2C (MEF2C) (26–28), together with fatty acid synthase (FASN), Atrogin-1 and myogenic differentiation 1 (Myod1) (29–31), modulating skeletal muscle health. Therefore, the protein levels of PPARγ, Foxo3a, and MEF2C as well as FASN, Atrogin-1, and Myod1 were detected in quadriceps. PPARγ level was increased while FASN level was decreased in EH group, compared with that in EC group, respectively (*P* < 0.05, Figures 3D,E). There were significant decreases of Foxo3a and Atrogin-1 protein levels in EH group compared with that in EC group (*P* < 0.05, Figures 3F,G). Inversely, both MEF2C and Myod1 protein levels had significant increases in EH group compared with that in EC group (*P* < 0.05, Figures 3H,I). Taken together, high dose of leucine supplementation for 12 weeks could influence muscle health through regulating AMPKα/SIRT1/PGC-1α in the aging mice.

### Leucine Supplementation Altered Microbiota Richness and Diversity

**Figure 4:**
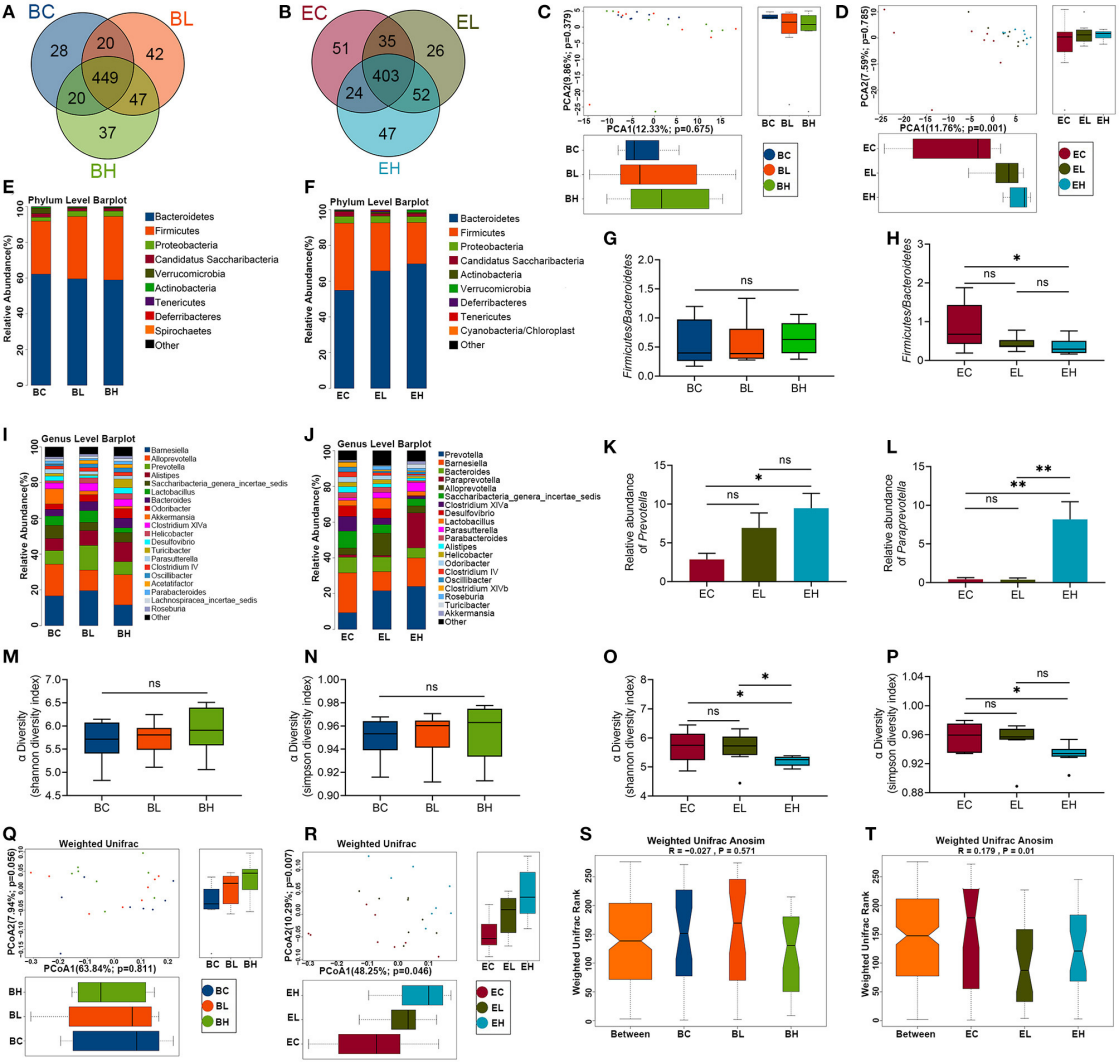
Microbiota richness and diversity before and after leucine supplementation. **(A,B)** OTU Venn diagrams in gut microbiota in the baseline and endpoint groups; **(C,D)** OTU principal component analysis (PCA) plots in the baseline and endpoint groups; **(E,F)** relative abundance of microbiota at phylum level in the baseline and endpoint groups; **(G,H)** the ratios of *Bacteroidetes* and *Firmicutes* in the baseline and endpoint groups; **(I,J)** relative abundance of top 20 genera in the baseline and endpoint groups; **(K,L)** relative abundance of *Prevotella* and *Paraprevotella* in endpoint groups; **(M,N)** Shannon and Simpson diversity index in the baseline groups; **(O,P)** Shannon and Simpson diversity index in the endpoint groups; **(Q,R)** principal coordinates analysis (PCoA) plots in the baseline and endpoint groups; **(S,T)** analysis of similarity (ANOSIM) analysis in the baseline and endpoint groups; ns, no significance; *P < 0.05; and **P < 0.01.

To examine whether the effects of leucine supplementation on muscle is related with gut microbiota, microbiota composition was detected in the feces of baseline and endpoint groups. There were 643 Operational Taxonomic Units (OTUs) and 638 OTUs in microbiota of baseline and endpoint groups, respectively. As shown in Figures 4A,B, the common OTUs were decreased after leucine supplementation. OTU principal component analysis (PCA) was applied to evaluate the intergroup differences of baseline and endpoint. The microbiota composition in the endpoint groups was rather obviously separated among the three groups than that of the baseline groups (Figures 4C,D), implying that leucine supplementation exerted influence on microbiota composition in the aging mice.

Relative abundance of microbiota at phylum level in the three groups before and after leucine supplementation was shown in Figures 4E,F. The ratio of *Firmicutes* and *Bacteroidetes* had no significant difference among the baseline groups (*P* > 0.05, Figure 4G). Nevertheless, the *Firmicutes*-*Bacteroidetes* ratio presented a significant decrease after high dose of leucine supplementation, compared with that of EC group (*P* < 0.05, Figure 4H). Besides, the top 20 genera were also displayed according to relative abundance in the baseline and endpoint groups (Figures 4I,J). *Barnesiella, Alloprevotella*, and *Prevotella* were the dominant bacterial genera among the baseline groups. Notably, *Prevotella* was increased to be the first dominant genus after 12 weeks of leucine intake (*P* < 0.05, Figure 4K), followed by *Barnesiella* and *Bacteroides*. Interestingly, *Paraprevotella* was markedly increased after high dose of leucine supplementation (*P* < 0.05, Figure 4L), compared with that of EC and EL groups.

Alpha diversity analysis was used to analyze the bacterial diversity in single sample to show the species richness and evenness. There were no significant difference of Shannon and Simpson index in the three groups before leucine supplementation (*P* > 0.05, Figures 4M,N). Whereas, both Shannon and Simpson index were significantly lower in the EH group than in the EC and EL group (*P* < 0.05, Figures 4O,P), suggesting that high-dose leucine supplementation for 12 weeks could decrease the richness and evenness of gut microbiota.

Beta diversity analysis was used to compare the differences of species diversity among the samples. A principal coordinates analysis (PCoA) presented that microbial community structure of different samples were obviously separated after leucine supplementation, while no difference was shown among the groups before leucine gavage (*P* > 0.05, Figures 4Q,R). Similarly, the differences of intergroup and intragroup were not significant in the baseline groups (*P* > 0.05), but the difference of intergroup was significantly greater than that of intragroup in the endpoint groups, as analysis of similarity (ANOSIM) analysis revealed (*P* < 0.05, Figures 4S,T). The results of PCoA and ANOSIM demonstrated that leucine supplementation could change the difference of microbiota diversity among the samples.

### More Differential Species and Metabolic Pathways Were Enriched After Leucine Supplementation

**Figure 5:**
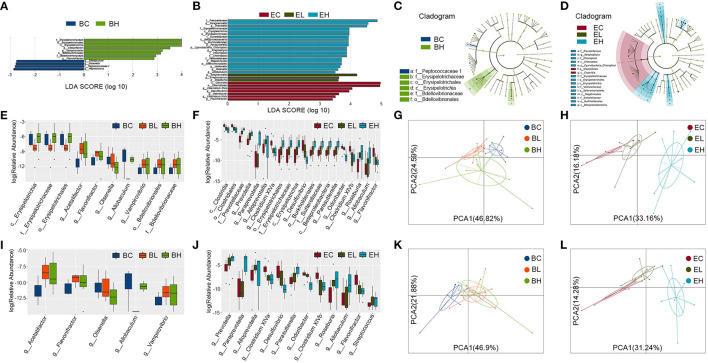
Differential species and metabolic pathways before and after leucine supplementation. **(A,B)** Linear discriminant analysis (LDA) effect size (LEfSe) analysis revealed 12 and 30 discriminative features at all the taxon levels in the baseline and endpoint groups (LDA > 2); **(C,D)** taxonomic cladograms in the baseline and endpoint groups; **(E,F)** Kruskal–Wallis rank sum test showed 10 and 30 (top 20) significantly different species at all the taxon levels in the baseline and endpoint groups; **(G,H)** the PCA plots at all taxon levels in the baseline and endpoint groups; **(I,J)** Kruskal–Wallis rank sum test revealed 5 and 15 (top 12) significantly different species at genus level in the baseline and endpoint groups; **(K,L)** the PCA plots at genus level in the baseline and endpoint groups.

To identify specific microbial communities associated with leucine supplementation, the compositions of gut microbiota in the baseline and endpoint groups were compared by using linear discriminant analysis (LDA) effect size (LEfSe) analysis. In total, LEfSe analysis revealed 12 discriminative features at all the taxon levels before leucine gavage (LDA > 2, *P* < 0.05, Figure 5A). After leucine supplementation, 30 discriminative features at all the taxon levels were identified (LDA > 2, *P* < 0.05, Figure 5B). Cladograms in the baseline and endpoint groups individually represented microbial communities playing vital effects in the groups (Figures 5C,D). In addition, the differential species identification in each group was also confirmed by Kruskal–Wallis rank sum test. There were 10 and 30 significantly different species enriched at all the taxon levels in the baseline and endpoint groups, respectively (*P* < 0.05, Figures 5E,F). The PCA plots at all the taxon levels revealed that there was no clear division among the baseline groups, whereas EH group was obviously separated from the EC and EL groups (Figures 5G,H). As shown above, the differential species identified by the two different analysis methods remained broadly consistent, suggesting the stability of gut microbiota profiling data.

Specific species at genus level was also distinguished by Kruskal–Wallis rank sum test. There were 5 and 15 significantly different species at genus level enriched in the baseline and endpoint groups, respectively (*P* < 0.05, Figures 5I,J, Supplementary Tables S1, S2). Particularly, *Prevotella, Paraprevotella, Alloprevotella, Parasutterella, Roseburia, Allobaculum*, and *Streptococcus* were significantly increased while *Clostridium* XlVa, *Desulfovibrio, Odoribacter, Clostridium* XlVb, and *Flavonifractor* were significantly decreased after leucine gavage. Nevertheless, *Acetatifactor, Allobaculum, Flavonifractor, Olsenella*, and *Vampirovibrio* were also significantly different in the baseline groups. The PCA plots at genus level demonstrated similar results with that at all the taxon levels described previously (Figures 5K,L). All the results above suggested that more differential species and metabolic pathways were enriched after leucine supplementation.

### Chronic High Dose of Leucine Supplementation Suppressed Pyruvate Fermentation to Propanoate I in Feces

**Figure 6:**
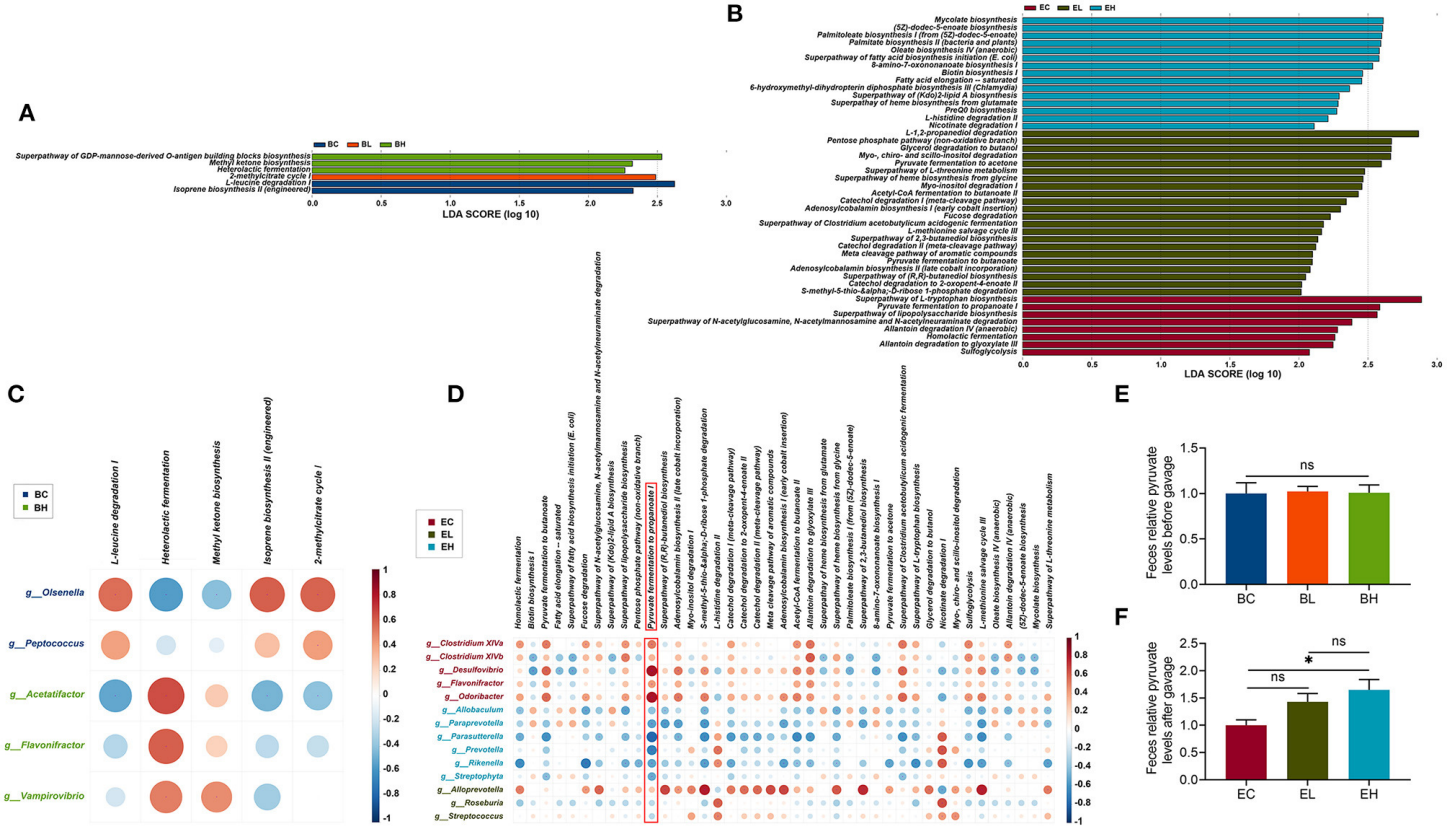
Chronic high dose of leucine supplementation suppressed pyruvate fermentation to propanoate I in feces. **(A,B)** Based on the MetaCyc metabolic pathways database, 6 and 45 differential metabolic pathways clustered in the baseline and endpoint groups (LDA > 2); **(C,D)** correlation networks between differential species at genus level and differential metabolic pathways; differential species seemed to more closely related to pyruvate fermentation to propanoate I in the endpoint groups among all the metabolic pathways. **(E)** The pyruvate levels in feces of the baseline groups; **(F)** the pyruvate levels in feces of endpoint groups; ns, no significance; *P < 0.05.

To characterize the functional alterations in the gut microbiota related to leucine supplementation, the functional composition profiles using 16S rRNA gene sequencing data were analyzed with PICRUSt2. As shown in Figures 6A,B, there were 6 and 45 differential metabolic pathways clustered in the baseline and endpoint groups (*P* < 0.05, Supplementary Tables S3, S4), based on the MetaCyc metabolic pathways database. The differential metabolic pathways were mainly classified into three categories, which were biosynthesis (fatty acid biosynthesis, amino acid biosynthesis, and enzyme cofactor biosynthesis), degradation/utilization/assimilation (amine and polyamine degradation, aromatic compound degradation, and secondary metabolite degradation), and generation of precursor metabolites and energy (fermentation). Taken together, more differential species and metabolic pathways were enriched in the feces after leucine supplementation.

To explore the effects of differential microbial communities on the metabolic pathways, a correlation network between differential species at genus level and differential metabolic pathways was constructed. There were five differential species and five differential metabolic pathways correlated in the baseline groups, while 14 differential species were associated with 41 differential metabolic pathways in the endpoint groups (*P* < 0.05, Figures 6C,D). More differential species in EC (*n* = 5) and EH (*n* = 6) groups correlated to differential metabolic pathways than that in EL (*n* = 3) groups. Moreover, the correlations between the metabolic pathways and differential species in EL group were not as strong as that in the EC and EH groups, which was probably driven by high dose of leucine gavage. Differential species showed closer relation to pyruvate fermentation to propanoate I in endpoint groups among all the metabolic pathways, which was positively correlated to differential species in EC group but was negatively associated with that in EH group. High-dose of leucine supplementation may inhibit pyruvate fermentation to propanoate I, indicating that pyruvate may increase after high-dose of leucine supplementation.

To examine the suppressive effects of leucine supplementation on pyruvate fermentation to propanoate I, the pyruvate levels were detected in the feces of baseline and endpoint groups. There was no difference of pyruvate levels in the feces of baseline groups (*P* > 0.05, Figure 6E), while the pyruvate levels in feces of EH group were significantly higher than that in EC group (*P* < 0.05, Figure 6F), demonstrating that high dose of leucine supplementation could surely increase pyruvate level in feces. Taken together, pyruvate fermentation to propanoate I was suppressed after high dose of leucine supplementation in the feces.

## Discussion

In present study, leucine supplementation for 12 weeks did not influence muscle mass and atrophy in 19-month-old mice but improved the histological and ultrasonographic changes in quadriceps. The previous studies with similar doses of leucine intake (675 and 1,350 mg/kg·d mostly) mainly focused on the short-term effects of leucine in the young or adult mice or rats, but changes in muscle mass and CSA were not so consistent in different studies for the variances of animal models, supplementation duration, and muscles (8, 23, 32–34). Besides, research on long- or short-term effects of leucine intake in the elderly animals and humans demonstrated long- or short-term leucine supplementation alone did not increase muscle weight and CSA while that in combination with exercise did (9, 22, 35, 36). Besides, a significant effect of leucine on muscle mass was shown in sarcopenic persons but not in the healthy subjects, and leucine supplementation on top of physical exercise could increase muscle mass (37). Exercise and sarcopenia may enhance the energy requirements to better promote leucine utilization, which may explain the negative results of leucine supplementation alone on muscle mass.

As results showed, chronic high dose of leucine activated AMPK/SIRT1/PGC-1α signaling pathway to regulate the expression of proteins related to lipid metabolism (PPARγ and FASN) (38, 39), muscle atrophy (Foxo3a and Atrogin-1) (29), and muscle protein synthesis (MEF2C and MyoD1) (31). Depressed protein level of Foxo3a and Atrogin-1 as well as the increased protein levels of MEF2C and MyoD1 indicated that leucine could enhanced MPS and alleviated atrophy in skeletal muscle after high dose of leucine gavage in the present study. PPARγ, well-known as a master regulator of adipocyte differentiation, whose depletion was completely devoid of adipose tissue (39). FASN functions as a central regulator of lipid metabolism for endogenous fatty acid synthesis (38). However, the increased protein level of PPARγ and decreased protein level of FASN were controversial after high dose of leucine supplementation in the present study. Surprisingly, PPARγ deletion was also demonstrated to cause insulin resistance in skeletal muscle (40) and impair muscle stem cells expansion and myogenesis after injury (41), indicating PPARγ has tissue-specific effects. Thus, the increased PPARγ levels in present study might have something to do with myogenesis.

Species richness, alpha and beta diversities were not different among the baseline groups, while lower gut microbiota richness and alpha diversity as well as different beta diversity were shown especially in group supplemented with high dose of leucine. Similarly, 6-week-old mice receiving 1.0% leucine also reduced the alpha and beta diversities of the gut microbiota (14). Additionally, the *Firmicutes*-*Bacteroidetes* ratio was decreased gradually as doses of leucine increased. As is known to all, *Firmicutes* and *Bacteroidetes* are the two groups of beneficial and dominant bacteria in the gut, increasing ratio of which was closely related to body-weight loss (42, 43). The previous studies also demonstrated that chronic leucine supplementation played a role of anti-obesity by lowering body weight and white adipose tissue weight (44, 45). Therefore, leucine supplementation in the present study may cause change in the gut microbiota associated with losing body weight or adipose tissue weight.

Predictably, there were differential species appearing among the baseline groups, probably due to the variances of individual, housing environment, sampling, or experiments. The first dominant genus *Prevotella* and the markedly-increased genus *Paraprevotella*, after leucine gavage in present study, belonging to *Bacteroidetes*, was also reported to enrich in high-physical-functioning older adults (18), both of which were also demonstrated to be the key negative bacteria in lipid increase (46–48). Moreover, *Prevotella* enrichment also had something to do with alleviating aging (49). Besides, other significantly increased genera *Alloprevotella, Parasutterella*, and *Allobaculum* and significantly reduced genera *Clostridium* XlVa, *Clostridium* XlVb, and *Desulfovibrio* were also previously reported to closely relate to obesity and diabetes (48, 50–52), which may contribute to age-related diseases (53). *Streptococcus* were positively associated with the muscle improvement outcomes (54). Moreover, the differential pathways in fatty acid biosynthesis as well as amino acid synthesis and degradation were enriched in the endpoint groups in present study, which was demonstrated to correlate to muscle metabolism (54). Therefore, gut microbiota may relate to the effects of leucine supplementation on promoting muscle health in aging mice.

**Figure 7:**
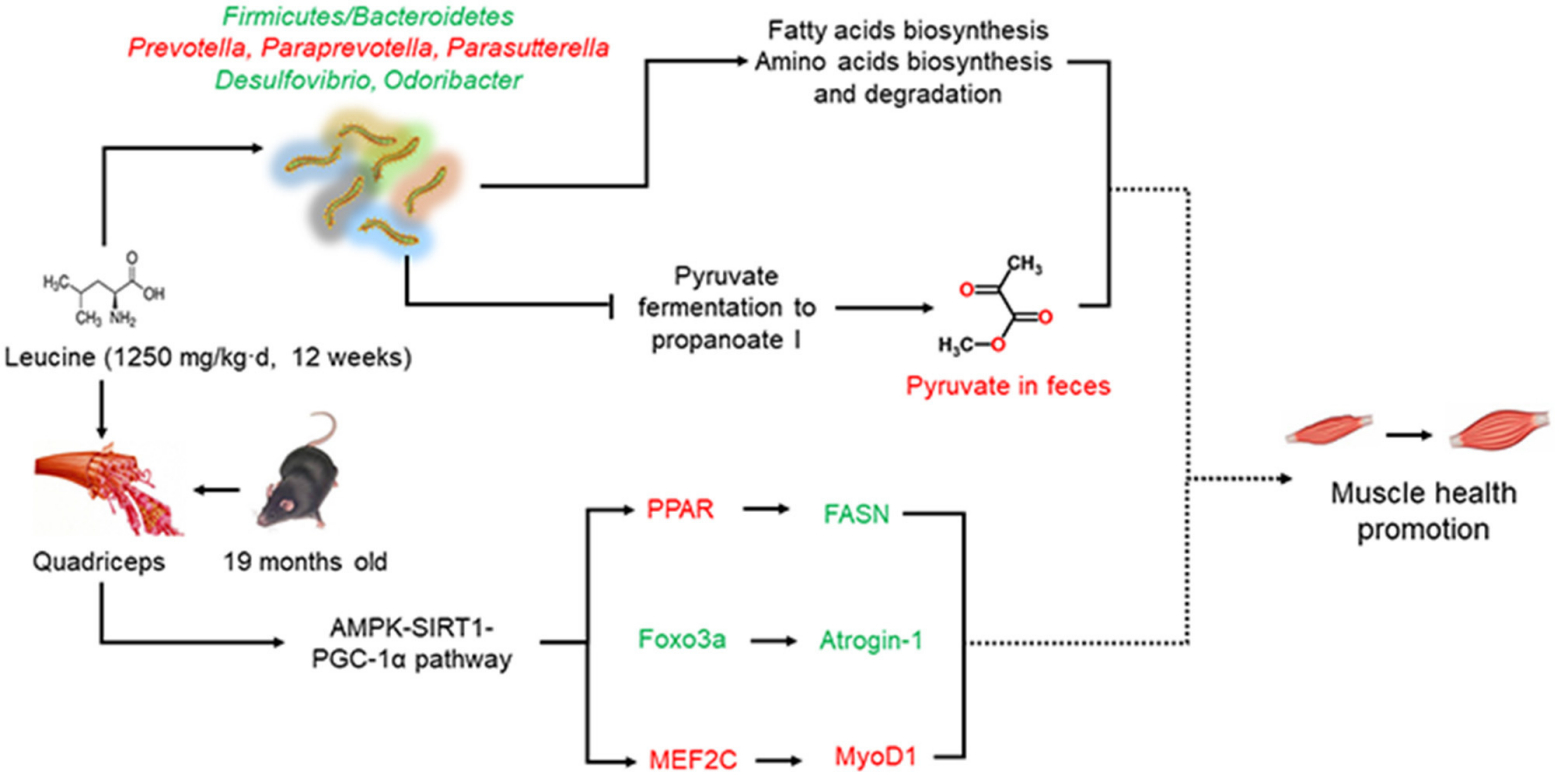
Schematic diagram of present work. Chronic high dose of leucine supplementation could promote muscle health *via* regulating AMPKα/SIRT1/PGC-1α. Besides, chronic high dose of leucine supplementation altered the composition and diversities of gut microbiota as well as increased pyruvate levels in the feces. Whether microbiota-related pyruvate level may correlate with the effects of chronic high dose of leucine supplementation on muscle health in the aging mice requires further study. Red indicates increased relative abundances or the expression levels while green denotes decreased relative abundances or expression levels.

Pyruvate fermentation to propanoate I, had stronger positive relation to differential genera in the EC group but negative correlation with differential genera in EH group, compared with other metabolic pathways. Pyruvate is a three-carbon intermediate compound between the glycolysis pathway that exerts an important role in oxidation and energy supply (55). Several published studies have demonstrated that pyruvate could induce weight loss and fat loss in the animals and humans by influencing energy metabolism (56–59). Remarkably, high dose of leucine supplementation induced significantly higher pyruvate levels in the feces in present study. Additionally, impaired mitochondrial pyruvate uptake led to strikingly decreased adiposity in skeletal muscle with complete muscle mass and strength retention, which highlighted the potential utility of modulating muscle pyruvate utilization to ameliorate obesity (60). Muscle aging was known to be accompanied with and exacerbated by adiposity (1). Furthermore, the pyruvate levels were reported to increase AMP/ATP and ADP/ATP ratios to subsequently activate AMPK and then control metabolism in aging (61). AMPK, a high sensitively nutrient and energy sensor, was also activated in this study in quadriceps. Besides, SIRT1 and PGC-1α, two famous molecular targets of anti-muscle aging, were regulated by AMPK phosphorylation (62, 63). Thus, we hypothesized that chronic high dose of leucine might be effective in advancing muscle health in aging mice through a potential pyruvate-associated AMPK/SIRT1/PGC-1α pathway (Figure 7), which requires more evidence to verify in the future.

In conclusion, chronic high dose of leucine supplementation could impact on skeletal muscle health *via* regulating AMPKα/SIRT1/PGC-1α in aging mice. The composition and diversities of gut microbiota were altered after leucine supplementation. Moreover, pyruvate fermentation to propanoate I were negatively associated with differential species and the pyruvate levels were significantly increased in feces after high dose of leucine supplementation, possibly related to AMPK/SIRT1/PGC-1α pathway in promoting muscle health. The present study shed light on the changes of pyruvate levels in feces, which would be a new focus in advancing muscle health by leucine supplementation in aging mice but requiring further investigation.

## Data Availability Statement

The datasets presented in this study can be found in online repositories. The names of the repository/repositories and accession number(s) can be found at: https://www.ncbi.nlm.nih.gov/, PRJNA688115.

## Ethics Statement

The animal study was reviewed and approved by Institutional Animal Care and Use Committee of Tongji Medical College, Huazhong University of Science and Technology.

## Author Contributions

YL and WY had primary responsibility for the final content and designed the analysis. YL, DL, and XZ analyzed the data. ZP, ZM, and RL provided statistical support. YL wrote the manuscript. All authors have read and approved the final manuscript.

## Funding

This work was financially supported by the An Qi Nutrition Fund (AF2017004) and the National Natural Science Foundation of China (NSFC82003463).

## Conflict of Interest

The authors declare that the research was conducted in the absence of any commercial or financial relationships that could be construed as a potential conflict of interest.

## Publisher's Note

All claims expressed in this article are solely those of the authors and do not necessarily represent those of their affiliated organizations, or those of the publisher, the editors and the reviewers. Any product that may be evaluated in this article, or claim that may be made by its manufacturer, is not guaranteed or endorsed by the publisher.
