# Supplementary material for: Pyruvate Might Bridge Gut Microbiota and Muscle Health in Aging Mice After Chronic High Dose of Leucine Supplementation
Source: Front Med (Lausanne). 2021 Nov 22;8:755803. doi: 10.3389/fmed.2021.755803 (PMC8645596; doi:10.3389/fmed.2021.755803)
Supplement: Supplementary file 1 [file Data_Sheet_1.docx]

***Supplementary Materials***

1. **Supplementary Methods**
   1. **16S rRNA gene amplification and sequencing**

Integrity and purity of the microbial genomic DNA was evaluated by 1% agarose gel electrophoresis and a NanoDrop 8000 spectrophotometer (Thermo Fisher Scientific, USA). DNA concentration was checked with Qubit® dsDNA HS Assay kit (Invitrogen). DNA sequencing libraries targeting the V3-V4 hyper-variable regions of 16S rRNA gene were prepared by PCR amplification using specific primers supplemented with Illumina sequencing adapters and sample-specific barcodes according to Illumina’s instructions (https://support.illumina.com/downloads/16s_metagenomic_sequencing_library_preparation.html). The primers used were 341F (5’-ACTCCTACGGGRSGCAGCAG) and 806R (5’-GGACTACVVGGGTATCTAATC). PCR reactions were performed using KAPA HiFi Hotstart Readymix PCR kit (KAPA Biosystems, USA). In detail, the 20 μL of PCR mixture consisted of 10 μL of 2$\times$ PCR Master Mix Solution, 5 μM of each primer (final concentration) and 10 ng of template DNA. The following thermal cycling conditions were used: initial denaturation at 95℃ for 180 s; followed by 28 cycles of 98℃ for 15 s, 58℃ for 20 s, 72℃ for 20 s, and a final extension at 72℃ for 150 s. After amplification, the libraries were purified with 2% agarose gel electrophoresis and AxyPrep DNA gel extraction kit (Axygen, USA). DNA concentration of each library was evaluated with Qubit® dsDNA HS Assay kit (Invitrogen). All libraries were pooled in equivalent amounts and sequenced using an Illumina HiSeq SBS V4 kit on the Hiseq 2500 platform with PE250 mode at Realbio Technology Co., Ltd (Shanghai, China).

- 1. **Bioinformatic analysis**

After trimming of barcodes and primers, all reads were checked and only reads met the following criteria were used in the next analyses: ambiguous base ‘N’ in a read ≤ 3; average base quality score of a read ≥ 20; length range of the reads was 220-500 bp. Quality-filtered reads were ranked according to their relative abundance, and singletons were discarded. They were then clustered into Operational Taxonomic Units (OTUs) based on 97% similarity threshold using de novo OTU picking protocol of UPARSE（<http://drive5.com/uparse/>) and chimeric sequences were identified and removed using Userach (version 7.0). OTUs were assigned to different taxonomic levels through RDP reference database (http://rdp.cme.msu.edu/) from phylum to genus with 80% confidence level. Relative abundance of OTUs and taxonomic phylotypes were calculated by normalization to the sum. OTU Flower Diagram was used to show the unique and shared OTUs among groups. Alpha diversity was assessed by Shannon index, beta diversity was assessed by Principal Coordinates Analysis (PCoA). Both alpha and beta diversity were calculated by Quantitative Insights Into Microbial Ecology (QIIME, <http://qiime.sourceforge.net/>). Linear discriminant analysis (LDA) Effect Size (LEfSe) algorithm was used to characterize bacterial phylotypes which were significantly different in abundance between groups (<http://huttenhower.sph.harvard.edu/galaxy/root/index>). Next, PICRUSt2 (phylogenetic investigation of communities by reconstruction of unobserved states, http://huttenhower.sph.harvard.edu/galaxy/root/index) was used to predict the functional profiles of gut microbiota.

1. **Supplementary tables**

**Table S1 Different species at genus level in baseline groups**

| taxonname | Mean (BC) | Mean (BH) | Mean (BL) | *P* value |
| --- | --- | --- | --- | --- |
| g__Acetatifactor | 0.000654 | 0.005842 | 0.005251 | 0.025371 |
| g__Allobaculum | 0.001161 | 0.000192 | 5.63E-06 | 0.014387 |
| g__Flavonifractor | 0.000642 | 0.001775 | 0.001561 | 0.041124 |
| g__Olsenella | 0.001008 | 0.00013 | 0.00071 | 0.013904 |
| g__Vampirovibrio | 9.01E-05 | 0.00058 | 0.000501 | 0.017118 |

**Table S2 Different species at genus level in endpoint groups**

| taxonname | Mean (EC) | Mean (EH) | Mean (EL) | *P* value |
| --- | --- | --- | --- | --- |
| g__Allobaculum | 0.000699 | 0.007352 | 0.000868 | 0.018387 |
| g__Alloprevotella | 0.011684 | 0.015397 | 0.050303 | 0.02948 |
| g__Clostridium XlVa | 0.026968 | 0.005436 | 0.011876 | 0.008493 |
| g__Clostridium XlVb | 0.008732 | 0.002366 | 0.002563 | 0.018808 |
| g__Desulfovibrio | 0.020202 | 0.001493 | 0.013876 | 0.002586 |
| g__Flavonifractor | 0.00569 | 0.001059 | 0.001284 | 0.029492 |
| g__Odoribacter | 0.009245 | 0.002191 | 0.007313 | 0.001488 |
| g__Paraprevotella | 0.004327 | 0.081801 | 0.003786 | 0.003183 |
| g__Parasutterella | 0.004191 | 0.020366 | 0.008636 | 0.011362 |
| g__Prevotella | 0.028732 | 0.094843 | 0.069373 | 0.038006 |
| g__Rikenella | 0 | 0.000811 | 0 | 0.000108 |
| g__Roseburia | 0.000473 | 0.005684 | 0.006417 | 0.028948 |
| g__Streptococcus | 5.07E-05 | 0.000242 | 0.000321 | 0.043534 |
| g__Streptophyta | 0 | 4.51E-05 | 0 | 0.010491 |
| g__Veillonella | 0 | 0.000203 | 0 | 0.037903 |

**Table S3 Differential metabolic pathways clustered in baseline groups**

| Superclasses | biomarker_name | enrich_group | LDA score | pvalue | description |
| --- | --- | --- | --- | --- | --- |
| Biosynthesis → Carbohydrate Biosynthesis → Sugar Biosynthesis → Sugar Nucleotide Biosynthesis → GDP-sugar Biosynthesis Superpathways | PWY-7323 | BH | 2.533281 | 0.049292 | Superpathway of GDP-mannose-derived O-antigen building blocks biosynthesis |
| Generation of Precursor Metabolites and Energy | PWY-7007 | BH | 2.318783 | 0.018921 | Methyl ketone biosynthesis |
| Generation of Precursor Metabolites and Energy → Fermentation → Fermentation to Short-Chain Fatty Acids → Fermentation to Lactate | P122-PWY | BH | 2.265589 | 0.046421 | Heterolactic fermentation |
| Degradation/Utilization/Assimilation → Carboxylate Degradation → Propanoate Degradation → 2-Methylcitrate Cycle | PWY0-42 | BL | 2.487922 | 0.022093 | 2-methylcitrate cycle I |
| Degradation/Utilization/Assimilation → Amino Acid Degradation → Proteinogenic Amino Acid Degradation → L-leucine Degradation | LEU-DEG2-PWY | BC | 2.62455 | 0.036243 | L-leucine degradation I |
| Biosynthesis → Secondary Metabolite Biosynthesis → Terpenoid Biosynthesis → Hemiterpene Biosynthesis | PWY-7391 | BC | 2.324728 | 0.017774 | Isoprene biosynthesis II (engineered) |

**Table S4 Differential metabolic pathways clustered in endpoint groups**

| Superclasses | biomarker_name | enrich_group | LDA score | pvalue | description |
| --- | --- | --- | --- | --- | --- |
| Biosynthesis → Fatty Acid and Lipid Biosynthesis → Fatty Acid Biosynthesis | PWYG-321 | EH | 2.610746 | 0.044047 | mycolate biosynthesis |
| Biosynthesis → Fatty Acid and Lipid Biosynthesis → Fatty Acid Biosynthesis → Unsaturated Fatty Acid Biosynthesis → (5Z)-dodecenoate Biosynthesis | PWY0-862 | EH | 2.608281 | 0.037161 | (5Z)-dodec-5-enoate biosynthesis |
| Biosynthesis → Fatty Acid and Lipid Biosynthesis → Fatty Acid Biosynthesis → Unsaturated Fatty Acid Biosynthesis → Palmitoleate Biosynthesis | PWY-6282 | EH | 2.600639 | 0.048195 | palmitoleate biosynthesis I (from (5Z)-dodec-5-enoate) |
| Biosynthesis → Fatty Acid and Lipid Biosynthesis → Fatty Acid Biosynthesis → Palmitate Biosynthesis | PWY-5971 | EH | 2.594914 | 0.028086 | palmitate biosynthesis II (bacteria and plants) |
| Biosynthesis → Fatty Acid and Lipid Biosynthesis → Fatty Acid Biosynthesis → Unsaturated Fatty Acid Biosynthesis → Oleate Biosynthesis | PWY-7664 | EH | 2.58221 | 0.035704 | oleate biosynthesis IV (anaerobic) |
| Biosynthesis → Fatty Acid and Lipid Biosynthesis → Fatty Acid Biosynthesis Superpathways | FASYN-INITIAL-PWY | EH | 2.581502 | 0.037161 | superpathway of fatty acid biosynthesis initiation (E. coli) |
| Biosynthesis → Other Biosynthesis → 8-Amino-7-oxononanoate Biosynthesis | PWY-6519 | EH | 2.534171 | 0.049663 | 8-amino-7-oxononanoate biosynthesis I |
| Biosynthesis → Cofactor, Carrier, and Vitamin Biosynthesis → Enzyme Cofactor Biosynthesis → Biotin Biosynthesis Superpathways | BIOTIN-BIOSYNTHESIS-PWY | EH | 2.462204 | 0.030273 | biotin biosynthesis I |
| Biosynthesis → Fatty Acid and Lipid Biosynthesis → Fatty Acid Biosynthesis | FASYN-ELONG-PWY | EH | 2.455412 | 0.029674 | fatty acid elongation -- saturated |
| Biosynthesis → Cofactor, Carrier, and Vitamin Biosynthesis → Carrier Biosynthesis → Single Carbon Carrier Biosynthesis → Folate Biosynthesis → 6-Hydroxymethyl-Dihydropterin Diphosphate Biosynthesis  Biosynthesis → Cofactor, Carrier, and Vitamin Biosynthesis → Vitamin Biosynthesis → Folate Biosynthesis → 6-Hydroxymethyl-Dihydropterin Diphosphate Biosynthesis | PWY-7539 | EH | 2.36745 | 0.032387 | 6-hydroxymethyl-dihydropterin diphosphate biosynthesis III (Chlamydia) |
| Biosynthesis → Cell Structure Biosynthesis → Lipopolysaccharide Biosynthesis | KDO-NAGLIPASYN-PWY | EH | 2.292166 | 0.042426 | superpathway of (Kdo)2-lipid A biosynthesis |
| Biosynthesis → Cofactor, Carrier, and Vitamin Biosynthesis → Enzyme Cofactor Biosynthesis → Heme Biosynthesis → Heme b Biosynthesis | PWY-5918 | EH | 2.284915 | 0.034133 | superpathay of heme biosynthesis from glutamate |
| Biosynthesis → Secondary Metabolite Biosynthesis | PWY-6703 | EH | 2.274309 | 0.036516 | preQ0 biosynthesis |
| Degradation/Utilization/Assimilation → Amino Acid Degradation → Proteinogenic Amino Acid Degradation → L-histidine Degradation | PWY-5028 | EH | 2.211271 | 0.02712 | L-histidine degradation II |
| Degradation/Utilization/Assimilation → Aromatic Compound Degradation → Nicotinate Degradation | PWY-722 | EH | 2.111516 | 0.007673 | nicotinate degradation I |
| Degradation/Utilization/Assimilation → Alcohol Degradation  Generation of Precursor Metabolites and Energy → Fermentation → Fermentation to Short-Chain Fatty Acids → Fermentation to Propanoate | PWY-7013 | EL | 2.867453 | 0.04066 | L-1,2-propanediol degradation |
| Generation of Precursor Metabolites and Energy → Pentose Phosphate Pathways | NONOXIPENT-PWY | EL | 2.671581 | 0.041482 | pentose phosphate pathway (non-oxidative branch) |
| Generation of Precursor Metabolites and Energy → Fermentation → Fermentation to Alcohols Superpathways | PWY-7003 | EL | 2.669384 | 0.007521 | glycerol degradation to butanol |
| Degradation/Utilization/Assimilation → Secondary Metabolite Degradation → Sugar Derivative Degradation → Sugar Alcohol Degradation  Superpathways | PWY-7237 | EL | 2.665207 | 0.005642 | myo-, chiro- and scillo-inositol degradation |
| Generation of Precursor Metabolites and Energy → Fermentation → Fermentation of Pyruvate | PWY-6588 | EL | 2.598549 | 0.035172 | pyruvate fermentation to acetone |
| Degradation/Utilization/Assimilation → Amino Acid Degradation → Proteinogenic Amino Acid Degradation → L-threonine Degradation  Superpathways | THREOCAT-PWY | EL | 2.477501 | 0.021764 | superpathway of L-threonine metabolism |
| Biosynthesis → Cofactor, Carrier, and Vitamin Biosynthesis → Enzyme Cofactor Biosynthesis → Heme Biosynthesis → Heme b Biosynthesis  Biosynthesis → Tetrapyrrole Biosynthesis → Porphyrin Compound Biosynthesis → Heme Biosynthesis → Heme b Biosynthesis  Superpathways | PWY-5920 | EL | 2.465713 | 0.048679 | superpathway of heme biosynthesis from glycine |
| Degradation/Utilization/Assimilation → Secondary Metabolite Degradation → Sugar Derivative Degradation → Sugar Alcohol Degradation | P562-PWY | EL | 2.458103 | 0.016245 | myo-inositol degradation I |
| Generation of Precursor Metabolites and Energy → Fermentation → Fermentation to Short-Chain Fatty Acids → Fermentation to Butanoate  Superpathways | PWY-5676 | EL | 2.431625 | 0.022148 | acetyl-CoA fermentation to butanoate II |
| Degradation/Utilization/Assimilation → Aromatic Compound Degradation → Catechol Degradation  Superpathways | PWY-5415 | EL | 2.344332 | 0.030501 | catechol degradation I (meta-cleavage pathway) |
| Biosynthesis → Cofactor, Carrier, and Vitamin Biosynthesis → Enzyme Cofactor Biosynthesis → Cobamide Biosynthesis → Cobamide de novo Biosynthesis → Adenosylcobamide Biosynthesis → Adenosylcobalamin de novo Biosynthesis  Superpathways | PWY-5507 | EL | 2.301849 | 0.002226 | adenosylcobalamin biosynthesis I (early cobalt insertion) |
| Degradation/Utilization/Assimilation → Carbohydrate Degradation → Sugar Degradation | FUCCAT-PWY | EL | 2.228465 | 0.026252 | fucose degradation |
| Generation of Precursor Metabolites and Energy → Fermentation → Fermentation of Pyruvate  Superpathways | PWY-6590 | EL | 2.179583 | 0.02712 | superpathway of Clostridium acetobutylicum acidogenic fermentation |
| Biosynthesis → Amino Acid Biosynthesis → Proteinogenic Amino Acid Biosynthesis → L-methionine Biosynthesis → L-methionine Salvage  Superpathways | PWY-7527 | EL | 2.166065 | 0.018316 | L-methionine salvage cycle III |
| Biosynthesis → Other Biosynthesis → Butanediol Biosynthesis  Superpathways | PWY-6396 | EL | 2.137585 | 0.0143 | superpathway of 2,3-butanediol biosynthesis |
| Degradation/Utilization/Assimilation → Aromatic Compound Degradation → Catechol Degradation  Superpathways | PWY-5420 | EL | 2.123177 | 0.011081 | catechol degradation II (meta-cleavage pathway) |
| Degradation/Utilization/Assimilation → Aromatic Compound Degradation → Benzoate Degradation  Superpathways | PWY-5430 | EL | 2.099489 | 0.008652 | meta cleavage pathway of aromatic compounds |
| Generation of Precursor Metabolites and Energy → Fermentation → Fermentation of Pyruvate  Generation of Precursor Metabolites and Energy → Fermentation → Fermentation to Short-Chain Fatty Acids → Fermentation to Butanoate | CENTFERM-PWY | EL | 2.09836 | 0.032631 | pyruvate fermentation to butanoate |
| Biosynthesis → Cofactor, Carrier, and Vitamin Biosynthesis → Enzyme Cofactor Biosynthesis → Cobamide Biosynthesis → Cobamide de novo Biosynthesis → Adenosylcobamide Biosynthesis → Adenosylcobalamin de novo Biosynthesis  Superpathways | P381-PWY | EL | 2.083097 | 0.009005 | adenosylcobalamin biosynthesis II (late cobalt incorporation) |
| Biosynthesis → Other Biosynthesis → Butanediol Biosynthesis  Superpathways | P125-PWY | EL | 2.049579 | 0.009256 | superpathway of (R,R)-butanediol biosynthesis |
| Degradation/Utilization/Assimilation → Aromatic Compound Degradation → Catechol Degradation | PWY-5419 | EL | 2.020497 | 0.011081 | catechol degradation to 2-oxopent-4-enoate II |
| Biosynthesis → Amino Acid Biosynthesis → Proteinogenic Amino Acid Biosynthesis → L-methionine Biosynthesis → L-methionine Salvage → S-methyl-5-thio-alpha-D-ribose 1-phosphate Degradation  Degradation/Utilization/Assimilation → Nucleoside and Nucleotide Degradation → S-methyl-5-thio-alpha-D-ribose 1-phosphate Degradation | PWY-4361 | EL | 2.016619 | 0.022315 | S-methyl-5-thio-&alpha;-D-ribose 1-phosphate degradation |
| Biosynthesis → Amino Acid Biosynthesis → Proteinogenic Amino Acid Biosynthesis → L-tryptophan Biosynthesis Superpathways | PWY-6629 | EC | 2.887543 | 0.002177 | superpathway of L-tryptophan biosynthesis |
| Generation of Precursor Metabolites and Energy → Fermentation → Fermentation of Pyruvate → Pyruvate Fermentation to Propanoate  Generation of Precursor Metabolites and Energy → Fermentation → Fermentation to Short-Chain Fatty Acids → Fermentation to Propanoate → Pyruvate Fermentation to Propanoate | P108-PWY | EC | 2.587457 | 0.013267 | pyruvate fermentation to propanoate I |
| Biosynthesis → Fatty Acid and Lipid Biosynthesis Superpathways | LPSSYN-PWY | EC | 2.567901 | 0.002795 | superpathway of lipopolysaccharide biosynthesis |
| Degradation/Utilization/Assimilation → Amine and Polyamine Degradation Superpathways | GLCMANNANAUT-PWY | EC | 2.384777 | 0.038006 | superpathway of N-acetylglucosamine, N-acetylmannosamine and N-acetylneuraminate degradation |
| Degradation/Utilization/Assimilation → Amine and Polyamine Degradation → Allantoin Degradation Superpathways | PWY0-41 | EC | 2.280292 | 0.002193 | allantoin degradation IV (anaerobic) |
| Generation of Precursor Metabolites and Energy → Fermentation → Fermentation to Short-Chain Fatty Acids → Fermentation to Lactate  Superpathways | ANAEROFRUCAT-PWY | EC | 2.261055 | 0.042109 | homolactic fermentation |
| Degradation/Utilization/Assimilation → Amine and Polyamine Degradation → Allantoin Degradation Superpathways | PWY-5705 | EC | 2.247123 | 0.025476 | allantoin degradation to glyoxylate III |
| Degradation/Utilization/Assimilation → Secondary Metabolite Degradation → Sugar Derivative Degradation → Sulfoquinovose Degradation | PWY-7446 | EC | 2.076487 | 0.00299 | sulfoglycolysis |
